# Supplementary material for: Severe Intrahepatic Cholestasis of Pregnancy—Potential Mechanism by Which Fetuses Are Protected from the Hazardous Effect of Bile Acids
Source: J Clin Med. 2023 Jan 12;12(2):616. doi: 10.3390/jcm12020616 (PMC9860676; doi:10.3390/jcm12020616)
Supplement: Supplementary file 1 [file jcm-12-00616-s001.zip › jcm-2036631-supplementary.pdf]

Supplementary Table S1. A comparison of neonatal outcome in singleton and twin pregnancies.

	Singleton (n=13)	Twins (n=10)	P value
Gestational age at delivery (median (weeks), 95% CI)	36.2 (34.6-37)	35.8 (33-37.1)	0.86
Birth weight percentile (median, %95 CI)	51 (36-75)	74.5 (31-88)	0.17
Maternal TBA (µmol/L , median, %95 CI)	72.25 (40.58-129.8)	61.91 (51.55-77.74)	0.36
TBA in umbilical vein (µmol/L, median, %95 CI)	24.52 (16.79-32.78)	17.11 (13.45-23.89)	0.057
bile acid decrease (%,median, 95% CI)	67.64 (48.72-78.96)	70.83 (66.09-77.76)	0.48
Meconium (%)	46.15	20	0.086

Supplementary Table S2. Prediction of meconium stained amniotic fluid.

	OR (Meconium)	95% CI
Singleton / twins[Twin]	0.1084	0.04 to 1.197
Gestational week at delivery	0.7413	0.3453 to 1.449
Birth weight percentile	1.015	0.9692 to 1.069
TBA maternal at delivery	1.003	0.9631 to 1.046
TBA level in umbilical vein	0.9922	0.8623 to 1.135
